# The non-invasive assessment of myocardial work by pressure-strain analysis: clinical applications

**DOI:** 10.1007/s10741-021-10119-4

**Published:** 2021-05-26

**Authors:** Dawud Abawi, Tommaso Rinaldi, Alessandro Faragli, Burkert Pieske, Daniel A. Morris, Sebastian Kelle, Carsten Tschöpe, Concetta Zito, Alessio Alogna

**Affiliations:** 1 Department of Internal Medicine and Cardiology, Charité – Universitätsmedizin Berlin, Campus Virchow-Klinikum, Augustenburgerplatz 1, 13353 Berlin, Germany; 2 Department of Clinical and Experimental Medicine, Cardiology Unit - University of Messina, Messina, 98100 Italy; 3 DZHK (German Centre for Cardiovascular Research), partner site Berlin, Germany; 4 Department of Internal Medicine/Cardiology, Deutsches Herzzentrum Berlin, Augustenburgerplatz 1, 13353 Berlin, Germany; 5 Berlin Institute of Health, Berlin, Germany; 6 Berlin Institute of Health Center for Regenerative Therapies (BCRT), Charité - University Medicine Berlin, Campus Virchow Clinic, Augustenburgerplatz 1, 13353 Berlin, Germany

**Keywords:** Non-invasive pressure-strain analysis, Myocardial work, Myocardial mechanics, Echocardiography, Strain, Speckle tracking

## Abstract

Pressure–volume (PV) analysis is the most comprehensive way to describe cardiac function, giving insights into cardiac mechanics and energetics. However, PV analysis still remains a highly invasive and time-consuming method, preventing it from integration into clinical practice. Most of the echocardiographic parameters currently used in the clinical routine to characterize left ventricular (LV) systolic function, such as LV ejection fraction and LV global longitudinal strain, do not take the pressure developed within the LV into account and therefore fall too short in describing LV function as a hydraulic pump. Recently, LV pressure-strain analysis has been introduced as a new technique to assess myocardial work in a non-invasive fashion. This new method showed new insights in comparison to invasive measurements and was validated in different cardiac pathologies, e.g., for the detection of coronary artery disease, cardiac resynchronization therapy (CRT)-response prediction, and different forms of heart failure. Non-invasively assessed myocardial work may play a major role in guiding therapies and estimating prognosis. However, its incremental prognostic validity in comparison to common echocardiographic parameters remains unclear. This review aims to provide an overview of pressure-strain analysis, including its current application in the clinical arena, as well as potential fields of exploitation.

## Introduction

The evaluation of left ventricular (LV) systolic function is of significant importance in all echocardiographic examinations. The most common parameter to assess systolic function is left ventricular ejection fraction (LVEF), whereas reduced LVEF is correlated with poor outcome [1–3]. However, LVEF is sensitive neither for the assessment of regional differences in myocardial function nor for diastolic dysfunction [4]. Strain analysis by speckle-tracking echocardiography (STE) has become more and more relevant in the evaluation of regional and global left ventricular function over the last years, primarily due to its ability to detect subclinical dysfunction in a variety of cardiac pathologies, including a condition of preserved LVEF [5–11]. A major limitation of myocardial strain-analysis lies in its load dependency, which can lead to misinterpretation of the actual myocardial contractility [12, 13].

**Fig. 1:**
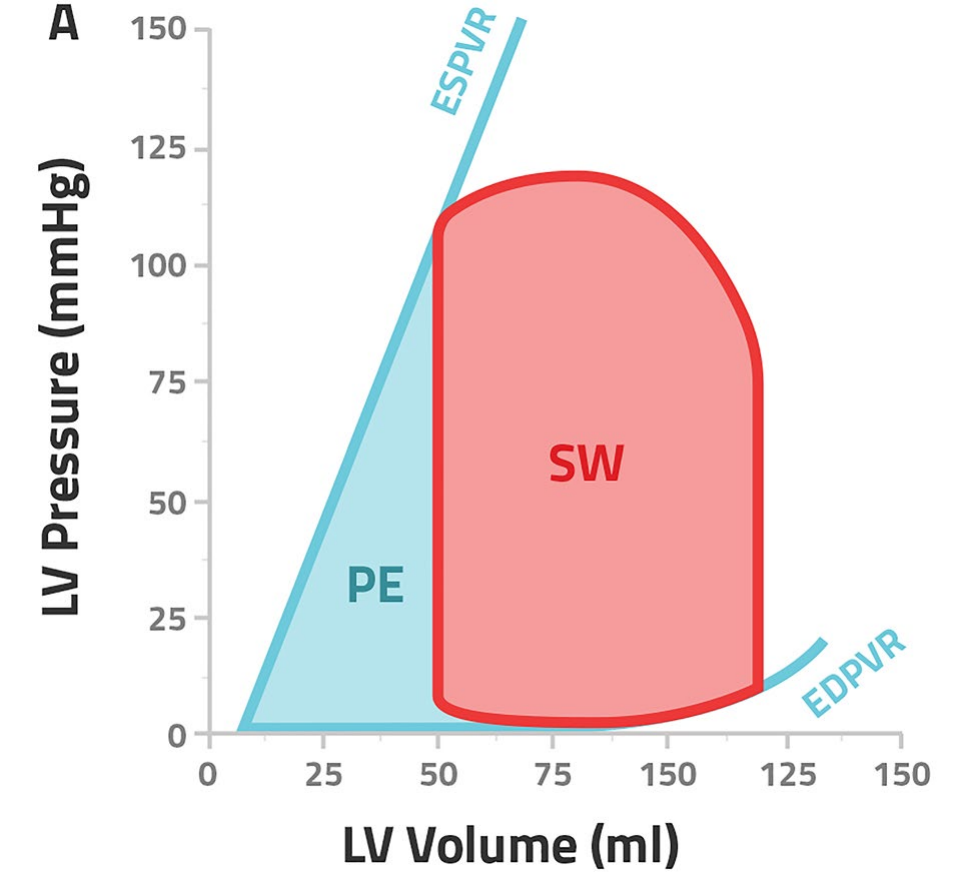
Pressure–volume analysis; the sum of the potential energy (PE) and stroke work (SW) describes the pressure volume area (PVA), ESPVR end-systolic pressure volume relationship, EDPVR end-diastolic pressure–volume relationship

Pressure–volume analysis, the gold standard in assessing ventricular function, delivers reasonably load-independent measures, as the end-systolic and end-diastolic pressure–volume relationships [14–17]. Furthermore, PV analysis gives insight into the ventricular-arterial coupling and offers information about myocardial energetics and efficiency. In terms of myocardial energetics, the total myocardial oxygen consumption per beat (MVO2) is described by the pressure–volume area (PVA), which is defined as the sum of stroke work (SW) and the potential energy (PE) [18–20]. Stroke work stands for the external work of the heart, meaning the energy which is required to eject blood into the vasculature system and is represented by the area of the pressure–volume loop [18]. PE is the potential myocardial work which is not liberated due to aortic valve closure and stored in the myofilaments (Fig. 1) [18]. Pressure–volume analysis has been proven to be an essential tool for the understanding of cardiovascular physiology and pathophysiology. However, due to its invasive nature and complexity, it has never been implemented into daily clinical practice.

Due to a further development in echocardiographic techniques including the implementation of volume and strain analysis, it became possible to estimate diastolic and systolic pressure volume relationships non-invasively [21, 22]. Recently, Russel et al. established a method to non-invasively assess myocardial work by obtaining pressure-strain-loops via STE based on a non-invasive approach to estimate left ventricular pressure (LVP) [22]. This review aims to provide an overview of the current state of research regarding this promising and holistic approach to non-invasively assess LV function.

### Theoretical background and first clinical validation

Whereas the pressure–volume loop describes the global myocardial work und function, also regional myocardial work has been studied in the form of pressure-length loops, obtained via micromanometers for the assessment of pressure, and implanted sonomicrometric crystals for the calculation of dimensions in animal models [23, 24].

The sonomicrometry technique, first introduced in 1956 by Rushmer et al. [25], has traditionally been used for the assessment of the dimensions of the heart [26, 27]. One of the first studies on the topic by Leraand et al. showed that ultrasonic elements sutured into the left myocardial wall of anesthetized dogs correctly measured local myocardial distances [28].

In analogy to the pressure–volume loop ability to reflect global myocardial oxygen consumption, Delhaas et al. could show that the regionally assessed stress-fiber strain area was not only able to reflect regional myocardial work but also regional oxygen demand [29].

Assessment of myocardial wall stress is difficult and not feasible in clinical practice. Studies by Skulstad and Urheim et al. could validate the invasively measured LV pressure as a surrogate of left ventricular wall stress [30, 31]. Furthermore, they demonstrated that regional myocardial work can be estimated from a combined measurement of invasively assessed LV pressure and myocardial strain by strain Doppler echocardiography (SDE) [30, 31]. Over a wide range of conditions, the SDE method correlated well with those measured by sonomicrometry [30, 31], leading to a more non-invasive approach of determining myocardial work.

The new approach by Russel et al. goes even a step further by assessing myocardial work completely non-invasively. They combined segmental strain curves by speckle tracking echocardiography with an estimated LV pressure curve where the systolic cuff pressure is used as a surrogate of LV peak pressure [22]. The method was validated in a variety of pathologies showing a good correlation to invasive measurements [5, 13, 22, 32–38]. Similar to the pressure–volume loop, non-invasive pressure-strain loops were shown to reflect regional myocardial oxygen consumption and metabolism validated by ^18^F-fluorodeoxyglucose positron emission tomography [22] (Table 1).

**Table 1:** Validation of non-invasive pressure-strain loops as a surrogate of myocardial work

Author	Title	Year	Journal	Number of patients	Results
Russel et al.	A novel clinical method for quantification of regional left ventricular pressure–strain loop area: a non-invasive index of myocardial work	2012	European Heart Journal	18	Non-invasive assessed regional LV pressure–strain loop area corresponds well with invasive measurements and reflects myocardial metabolism
Russel et al.	Assessment of wasted myocardial work: a novel method to quantify energy loss due to uncoordinated left ventricular contractions	2013	American Journal of Physiology-Heart and Circulatory Physiology	48	Energy loss caused by uncoordinated contractions can be measured as the LV wasted work ratio non-invasively
Hubert et al.	Estimation of myocardial work from pressure–strain loops analysis: an experimental evaluation	2018	European Heart Journal—Cardiovascular Imaging	9	The non-invasive assessment of LV myocardial work by pressure strain analysis correlates well with invasive measurements
Cauwenberghs et al.	Area of the pressure-strain loop during ejection as non-invasive index of left ventricular performance: a population study	2019	Cardiovascular Ultrasound	356	Integration of the LV pressure–strain loop during ejection might be a useful tool to non-invasively evaluate sex-specific and interdependent effects of preload and afterload on LV myocardial performance
Manganaro et al.	Echocardiographic reference ranges for normal non-invasive myocardial work indices: results from the EACVI NORRE study	2019	European Heart Journal Cardiovascular Imaging	226	The NORRE study provides reference ranges for non-invasive MW indices
Manganaro et al.	Correlation between non-invasive myocardial work indices and main parameters of systolic and diastolic function: results from the EACVI NORRE study	2019	European Heart Journal Cardiovascular Imaging	226	Non-invasively assessed MW indices correlate well with common 2DE measures of myocardial systolic function and strain
Galli et al.	Echocardiographic reference ranges for myocardial work in healthy subjects: A preliminary study	2019	Echocardiography	115	Referral ranges of GCW, GWI, GWW, and GWE are not dependent on age

*MW* myocardial work, *2DE* two-dimensional echocardiography, *GCW* global myocardial constructive work, *GWI* global myocardial work index, *GWW* global myocardial wasted work, *GWE* global myocardial work efficiency

### Segmental work calculation

In general, a vendor-specific module (EchoPAC Version 202, GE) is necessary to assess non-invasive myocardial work by combining left ventricular strain data obtained via speckle‐tracking echocardiography with a non-invasively estimated LV pressure curve.

Practically, LV global longitudinal strain (GLS) has to be estimated by processing two-dimensional grayscale images acquired in the standard apical two‐, three‐, and four‐chamber views at similar heart rate, depth, and a frame rate between 38 and 80 frames/s with an off-line dedicated software (Automated Functional Imaging; EchoPAC®, Version 202, GE).

The novelty of integrating non-invasive LV pressure is based on the creation of a normalized left ventricular pressure curve which was obtained by assembling invasive LVP measurements from patients in different conditions of inotropy [22]. All invasive LVP measurements were normalized to equal durations of isovolumetric contraction, ejection phase, and isovolumetric relaxation as well as the amplitudes of left ventricular peak pressure [22].

To then establish each patient’s specific LVP curve, the normalized LVP curve has to be adjusted according to the patient’s valvular event times assessed via echocardiography and the measured systolic cuff pressure, as a surrogate for LV peak pressure [22].

**Fig. 2:**
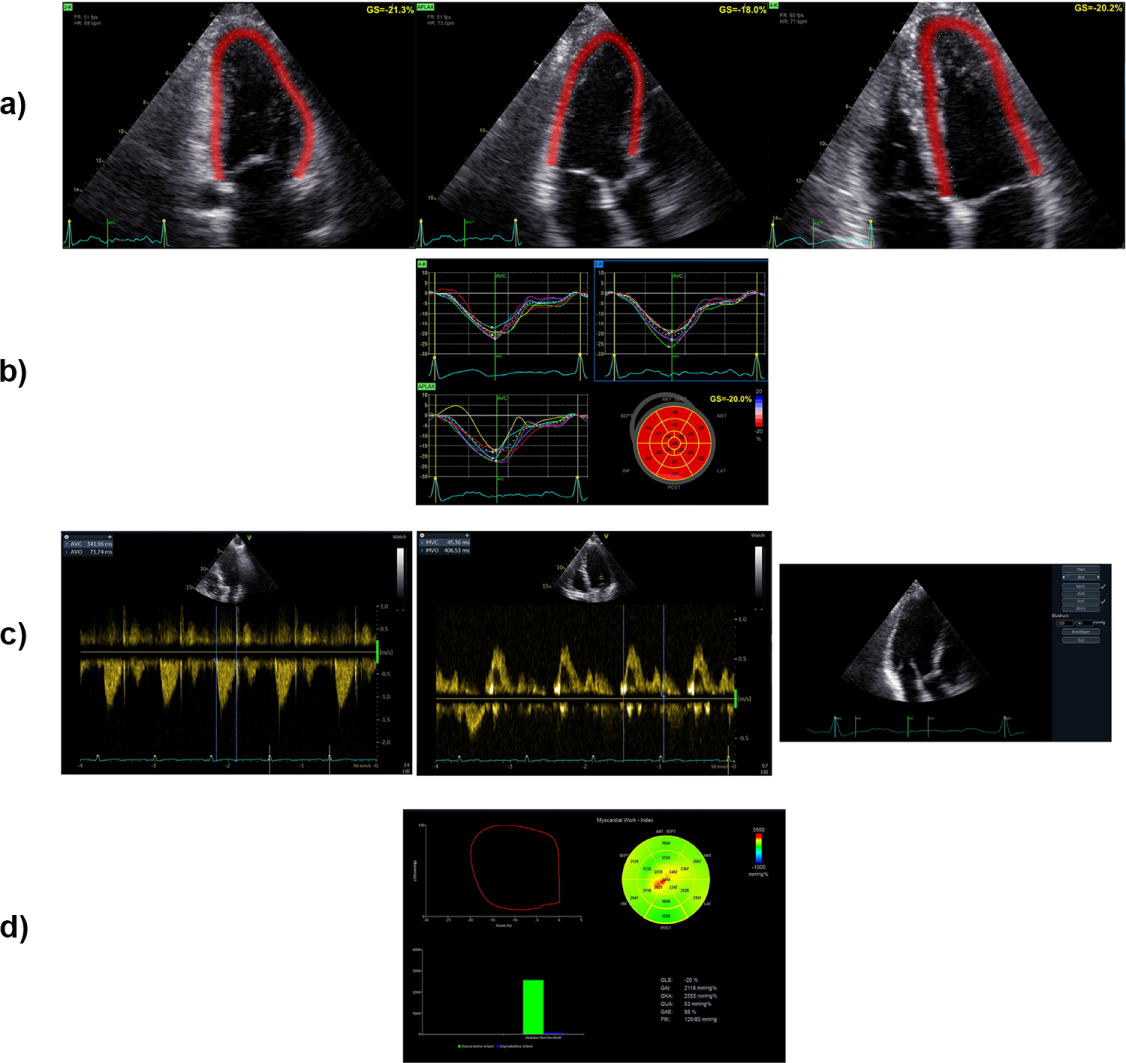
Practical assessment of myocardial work. **a** Global longitudinal strain is calculated through two-dimensional grayscale images acquired in the standard apical two-, three- and four-chamber views. **b** Visualization of the calculated strain measurements. **c** Determination of valvular event timings using pulsed-wave Doppler or ECG-triggering and insertion of systolic and diastolic blood pressure levels for myocardial work calculation. **d** Visualization of myocardial work via the pressure-strain loop

To determine valvular events, event timing can be derived by ECG-triggering or Doppler echocardiography. Electrical and mechanical phases should be aligned and can be manually adjusted. It is recommended to assess the blood pressure at the time of the examination (Fig. 2).

**Fig. 3:**
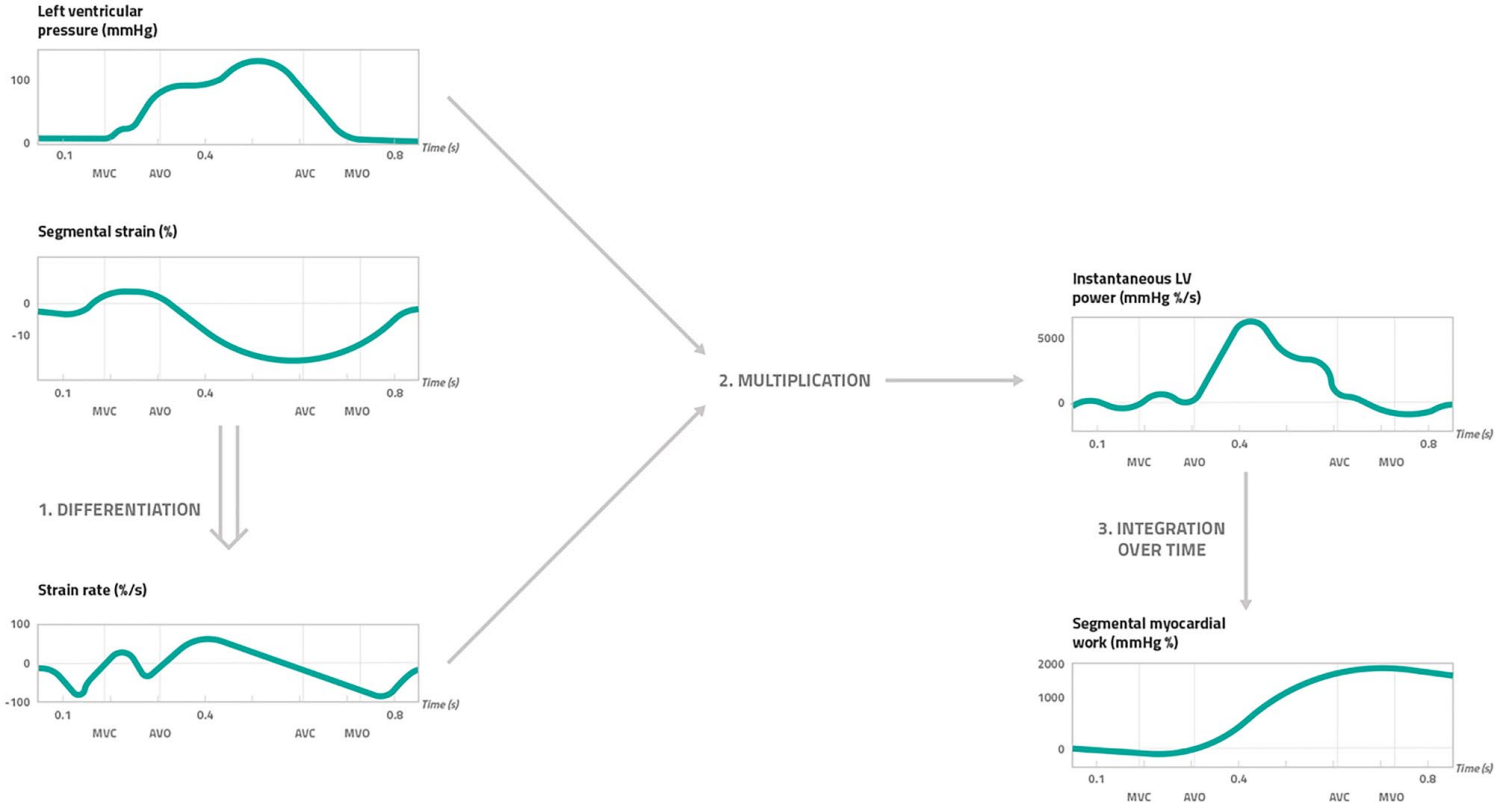
Calculation of segmental myocardial work by pressure strain-analysis. Myocardial work by pressure strain analysis is calculated as a function of time throughout the cardiac cycle. The calculational steps include (1) the differentiation of the segmental strain (%) in order to obtain the strain rate (%/s) which was then (2) multiplied with the left ventricular pressure (mmHg). This results in a measure of instantaneous LV power (mmHg %/s). To finally obtain segmental myocardial work over time (mmHg %), (3) the instantaneous LV power has to be integrated over time. MVC mitral valve closure, AVO atrial valve opening, AVC atrial valve closure, MVO mitral valve opening

A 17‐segment model is considered for the calculation of segmental myocardial work. However, segmental myocardial work is not assessed by calculating the area of each segmental pressure strain loop, rather it is calculated as a function of time for the duration of the whole cardiac cycle [32]. In particular, according to Russel et al. the strain recordings have to be differentiated to acquire the corresponding strain rate, which is then multiplied with the instantaneous LV pressure, resulting in a measure of power [32]. To finally obtain myocardial work as a function of time, instantaneous power also has to be integrated over time (Fig. 3).

**Fig. 4:**
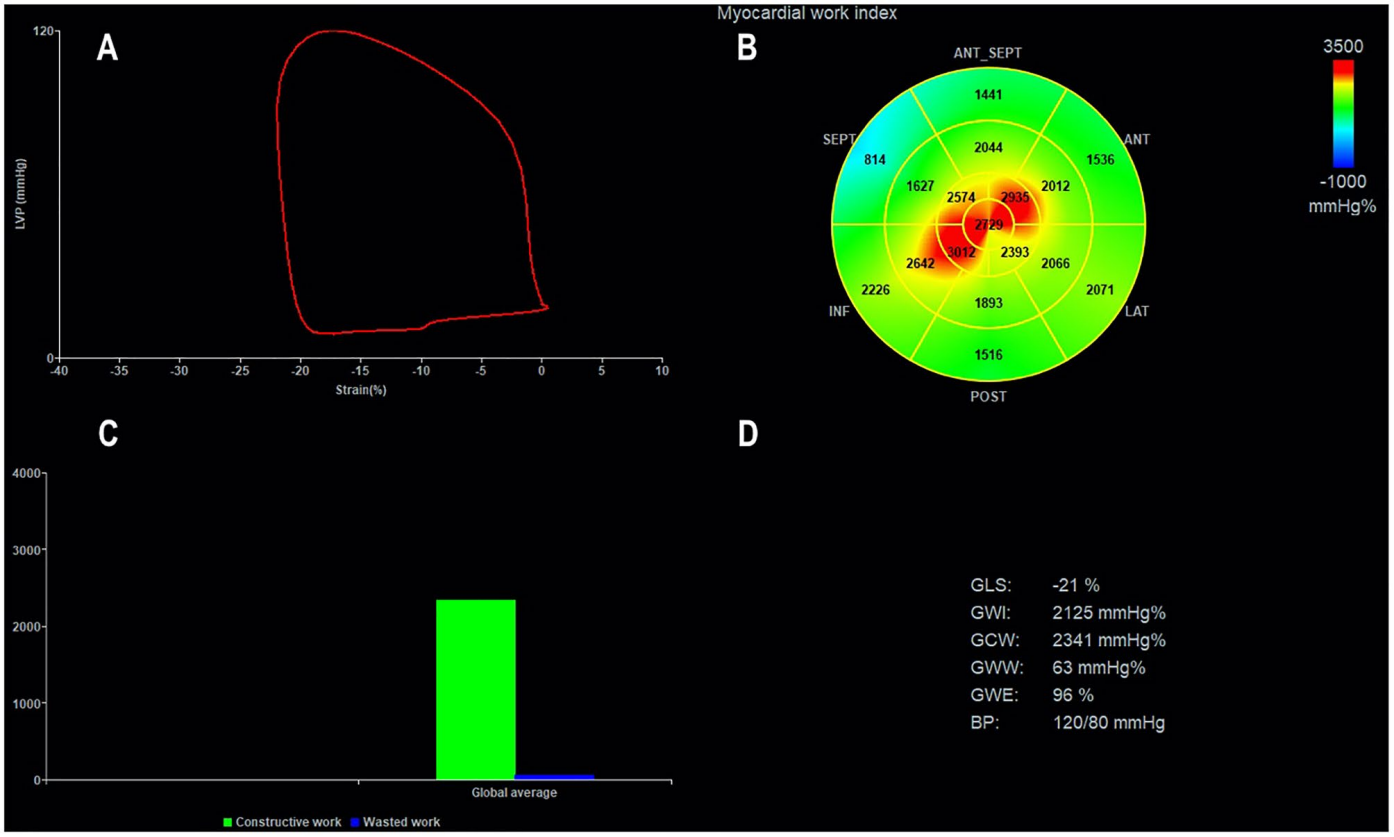
Measurement of myocardial work indices by 2D echocardiography. **A** Left ventricular pressure–strain loop. **B** Bull’s eye of global work index (GWI). **C** Bar graphs depicting global constructive work (GCW) and global wasted work (GWW). **D** Results from myocardial work analysis

Global values of myocardial work are derived from the average of all segmental myocardial work values. Further parameters describe the relationship between positive and negative myocardial work according to their occurrence during the cardiac cycle (Fig. 4):

- Constructive myocardial work (GCW): positive segmental work performed during myocardial shortening in systole and negative segmental work during lengthening in isovolumic relaxation
- Wasted myocardial work (GWW): negative segmental work performed during lengthening in systole and positive segmental work performed during myocardial shortening in isovolumic relaxation
- Myocardial work efficiency (GWE): relationship between constructive work and the sum of constructive and wasted work

## Methods

For this literature review, we searched MEDLINE (PubMed) and Embase, while clinicaltrials.gov was searched for ongoing clinical studies. The following search terms included in the title were used: “cardiac work,” “constructive work,” “global longitudinal strain,” “global work index,” “myocardial work,” “myocardial work efficiency,” “non-invasive myocardial work,” in combination with “pressure-strain loops” in the title or abstract. Relevance and credibility of all the sources were considered, and the final decision on inclusion was reached through a consensus of the following screening authors: DA, TR, AF, and AA. Review articles, case reports, comments, and author replies were excluded. The cited references were published within the last 8 years. A narrative synthesis with a tabulation system was used to analyze studies for their diverse research designs, methods, and implications.

### Clinical applications

**Fig. 5:**
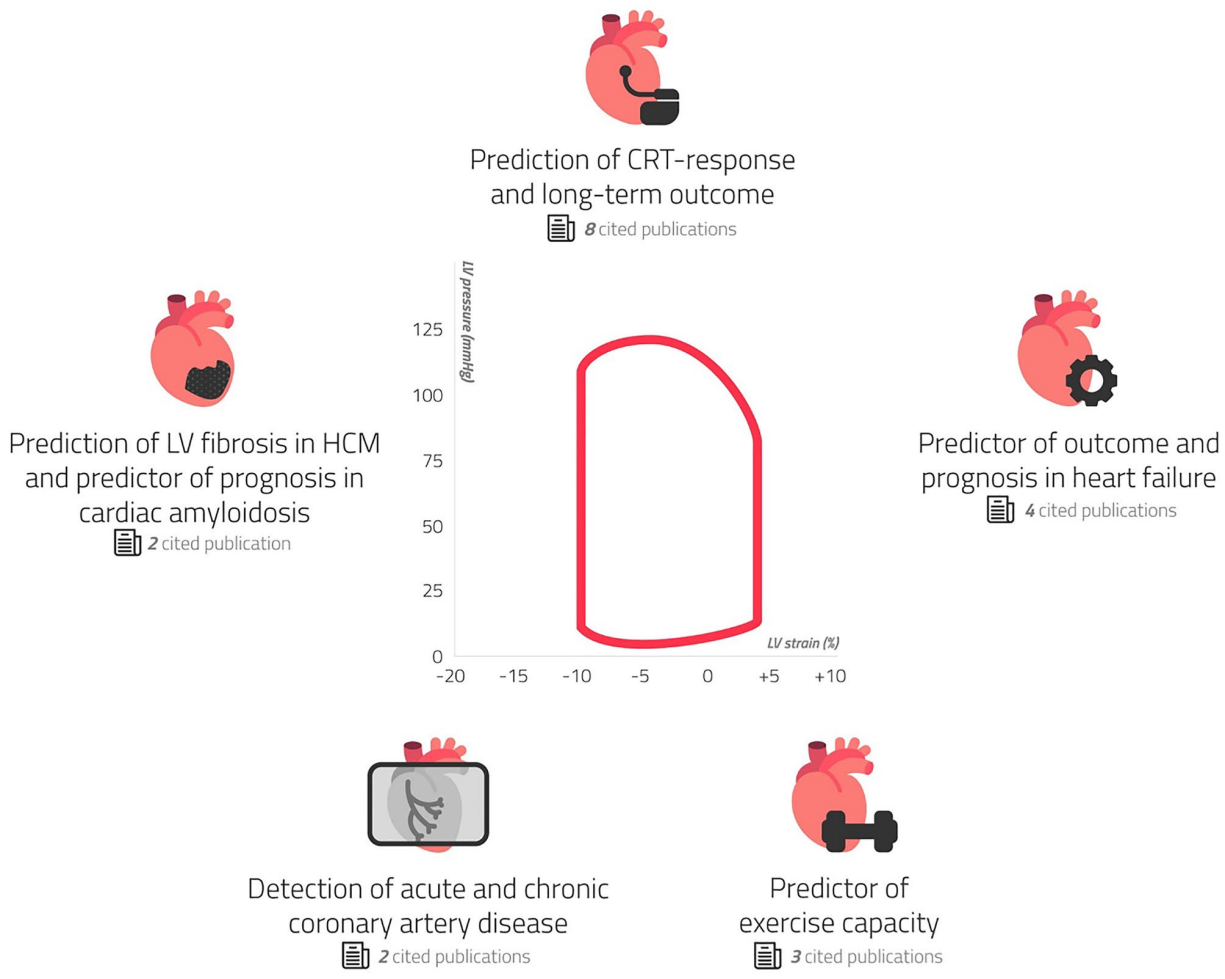
Current scientific articles on myocardial work divided according to different clinical subtopics

In the present review, we focused on the state of the art regarding non-invasive myocardial work assessment in several clinical fields: in CRT recipients, in patients with ischemic cardiac disease, mitral valve repair, heart failure with reduced (HFrEF) and those with heart failure and preserved ejection fraction (HFpEF) or HFpEF-like syndromes (hypertrophic cardiomyopathy, cardiac amyloidosis) (Fig. 5).

Furthermore, we searched for ongoing clinical trials focusing on non-invasive myocardial work assessed by pressure-strain loops.

### Cardiac resynchronization therapy

Cardiac resynchronization therapy (CRT) is an effective therapy that aims to restore mechanical efficiency to the failing LV by resynchronizing the contraction of the left and right ventricle, resulting in a reduction of both morbidity and mortality [39]. According to the 2016 ESC guidelines for the diagnosis and treatment of heart failure, CRT is taken into account with a class I a recommendation in symptomatic patients with heart failure (HF) in sinus rhythm with a QRS duration ≥ 150 ms and left bundle branch block (LBBB) as well as with an LVEF ≤ 35% despite optimal medical therapy (OMT); in patients with HFrEF regardless of their NYHA class who indicate for ventricular pacing and high degree AV block (including patients with atrial fibrillation) [40].

However, the number of patients who do not respond to CRT remains high (30–35%) [41]. Estimating CRT response is difficult as there is a lack of a globally accepted CRT response definition. Mostly, CRT response is identified by a ≥ 10–15% reduction of LV end-systolic volume (LVESV), assessed by echocardiography 6 months after device implantation, as a sign of LV reverse remodeling [42]. Current patient selection criteria utilize the surface 12-lead ECG to identify electromechanical delay; here, the presence of left bundle branch block morphology is considered to be a predictor of response to CRT [39]. However, it is becoming increasingly clear that QRS duration is an inadequate predictor of CRT response, since LV dyssynchrony is not uncommon in patients with a narrow QRS, proving how electrical dyssynchrony does not always correlate to mechanical delay [43]. In such cases, mechanical dyssynchrony may be, in fact, the result of abnormalities in regional contractility of the LV and loading conditions which can be better assessed through further imaging techniques, rather than ECG. However, although some echocardiographic dyssynchrony parameters have proven to be valuable in predicting CRT response in numerous, single-center studies [44, 45], the multi-center PROSPECT trial showed how such predictors are burdened with low sensitivity and specificity [42]. Therefore, as of today, no imaging technique is accepted for the identification of CRT responders, as also pointed out by 2016 ESC heart failure guidelines, which discourage the use of echocardiographic dyssynchrony criteria for the selection of patients [40]. Lastly, some clinical evaluations have also been proposed to detect CRT responders: male sex and ischemic etiology were shown as good predictors to less favorable CRT response [46, 47]. Nonetheless, when they were considered together with baseline LV volumes, they proved to be no longer independent variables [48]. Regarding the use of pressure-strain analysis, Galli et al. could show that GCW and GWW were associated with a positive response to CRT [36, 38]. GCW and GWW at baseline were significantly higher in CRT-responders in comparison to non-responders. After 6 months of follow up, CRT responders presented an increase in GCW and a reduction in GWW associated with LV reverse remodeling. Based on the findings of Russel et al. indicating that pressure strain measurements reflect cardiac metabolism, Galli et al. hypothesized that higher GCW at baseline represents a higher contractile reserve and is therefore able to predict positive CRT response, whereas decreased amount of GCW could be associated with reduced myocardial viability, limiting the beneficial effects of CRT [22, 36, 49, 50]. Furthermore, Ciampi et al. previously showed that the existence of contractile reserve assessed by dobutamine stress echocardiography is associated with a better prognosis, which is independent of the presence of LV dyssynchrony, as LV stimulation recruits viable myocardium [50]. There are several methods for the assessment of myocardial viability available, e.g., stress echocardiography, cardiac magnetic resonance, and nuclear imaging. Non-invasive estimation of myocardial work by pressure-strain analysis could provide a simple, easily accessible and cost-saving additive method. Additionally, Galli et al. confirmed a predictive value of GWW for positive CRT response, postulating that GWW might stand for a recruitable energy waste which could be significantly reduced in positive CRT responders [36]. The combination of GCW > 1057 mm Hg% and GWW > 364 mm Hg% showed very good specificity with low sensitivity [36]. Hence, myocardial work indices can be used to identify CRT responders, but patients might also benefit from CRT if GCW and GWW are below the mentioned cut-off values [36]. However, given the existence of numerous independent mechanisms influencing CRT response, the study suggests that the combination of clinical, electrocardiographic, and echocardiographic data in particular might be more useful to detect CRT responders [50–55]. In an earlier study by Vecera et al., also septal wasted work, especially in combination with the wall motion score index (WMSI), strongly predicted CRT response [56]. These results were confirmed in a study by Zhu et al. using a 3D echocardiography and 3D speckle tracking imaging analysis to simultaneously obtain left ventricular global and segmental principal strain and volume. Here, the measurement of baseline septal myocardial WW helped to enable a better patient selection for CRT (the more septal wasted at baseline, the higher probability of response to CRT was achieved) [57]. Also, there are preliminary data indicating that preserved GWE and GCW before CRT are associated with improved long-term outcome [37, 58]. Recently, Duchenne et al. could demonstrate that the acute redistribution of regional myocardial work after CRT device implantation from the lateral to the septal wall appeared to be strongly related to long-term reverse remodeling. Proper patient selection should consider the presence of loading inhomogeneities for a successful resynchronization therapy [59] (Table 2). In a study by Kostyukevich et al., CRT responders had significantly larger septal WW and lateral CW compared to non-responders at baseline. Plus, on multivariate analysis, baseline lateral CW was independently associated with CRT response (CW > 881 mm Hg%) [60]. Moreover, CRT responders demonstrated a significant improvement in septal CW and WW as well as a decrease and an increase in lateral CW and WW respectively [60].

**Table 2:** Non-invasive myocardial work and cardiac resynchronization therapy (CRT)

Author	Title	Year	Journal	Number of patients	Results
Vecera, J. et al.	Wasted septal work in left ventricular dyssynchrony: a novel principle to predict response to cardiac resynchronization therapy	2016	European Heart Journal Cardiovascular Imaging	21	Wasted work in the septum together with LV wall motion score can give a better prediction of response to CRT
Galli et al.	Role of myocardial constructive work in the identification of responders to CRT	2018	European Heart Journal Cardiovascular Imaging	97	Patients with higher GCW show a positive response to CRT
Galli et al.	Value of myocardial work estimation in the prediction of response to cardiac resynchronization therapy	2018	Journal of the American Society of Echocardiography	97	WW and CW are useful indexes to better understand CRT response and mechanisms of dyssynchrony
Van der Bijl et al.	Prognostic implications of global, left ventricular myocardial work efficiency before cardiac resynchronization therapy	2019	European Heart Journal Cardiovascular Imaging	153	Lower baseline GLVMWE predicts improved long-term outcome
Galli et al.	Myocardial constructive work and cardiac mortality in resynchronization therapy candidates	2019	American Heart Journal	166	CW predicts outcome in CRT candidates
Zhu et al.	The value of left ventricular strain–volume loops in predicting response to cardiac resynchronization therapy	2019	Cardiovascular Ultrasound	60	Measurement of baseline septal WW can improve patient selection for CRT
Kostyukevich et al.	Regional left ventricular myocardial work indices and response to cardiac resynchronization therapy	2020	JACC: Cardiovascular Imaging	168	CRT responders show larger septal WW and lateral CW at baseline and demonstrate a significant improvement in septal CW and WW as well as a decrease and an increase in lateral CW and WW respectively
Duchenne et al.	Acute redistribution of regional left ventricular work by cardiac resynchronization therapy determines long-term remodeling	2020	European Heart Journal	130	The acute redistribution of regional myocardial work between the septal and lateral wall is an important predictor of CRT response

*CRT* cardiac resynchronization therapy, *GCW* global myocardial constructive work, *CW* constructive work, *WW* wasted work, *GLVMWE* global left ventricular myocardial work efficiency

In conclusion, even though pressure strain loops and related indexes cannot be used on their own to assess CRT candidates, they represent a novel method that shows promising results in the prediction of CRT response and long-term outcomes.

### Ischemic heart disease

In patients with non-ST-elevation myocardial infarction (NSTEMI), Boe et al. could show that the non-invasively estimated myocardial work index (MWI) was able to detect acute coronary occlusion, being superior to all other echocardiographic parameters used, including strain analysis [33]. Patients with NSTEMI represent a very heterogonous group in which timing of invasive therapy is not well defined [61, 62]. The identification of acute coronary occlusion in patients with NSTEMI could be beneficial in identifying patients who would benefit from direct or early revascularization [33].

In a further study, Edwards et al. validated that non-invasively assessed global myocardial work at rest allowed to detect subclinical coronary artery disease (CAD) in patients with preserved LVEF and no regional wall motion abnormalities (RWMAs) [34]. Interestingly, global myocardial work was even more sensitive in detecting subclinical coronary disease than global longitudinal strain (GLS), also in patients only suffering from a single-vessel disease [34]. GLS has a predominant contribution from the longitudinal arranged endocardial layer and therefore detects early ischemia-induced cardiac dysfunction since the subendocardium is more sensitive for reduced perfusion [63–70]. Other echocardiographic parameters that represent radial thickening as LVEF or RWMAs are less sensitive for the early derangement of myocardial function caused by ischemia [71, 72]. Here, global myocardial work performed even better than GLS underlining the sensitivity of myocardial work for myocardial oxygen consumption which seems to be reduced in early stages of CAD where LVEF is still preserved, and RWMAs are still absent [34].

In a retrospective analysis by El Mahdiui et al., a reduced myocardial work efficiency could be confirmed in patients with recently revascularized ST-elevation myocardial infarction and patients with heart failure with reduced ejection fraction [73]. However, there was no alteration of myocardial work efficiency in patients with no structural heart disease or patients presenting typical cardiovascular risk factors in comparison to healthy individuals [73] (Table 3).

**Table 3:** Ischemic heart disease and further applications of non-invasive myocardial work

Author	Title	Year	Journal	Number of patients	Results
Boe et al.	Non-invasive myocardial work index identifies acute coronary occlusion in patients with non-ST-segment elevation-acute coronary syndrome	2015	European Heart Journal Cardiovascular Imaging	150	Deteriorated MWI in patients with NSTEMI detects acute coronary occlusion superior to all other parameters
Edwards et al.	Global myocardial work is superior to global longitudinal strain to predict significant coronary artery disease in patients with normal left ventricular function and wall motion	2019	Journal of the American Society of Echocardiography	115	Non-invasive global MW at rest is better than GLS at identifying significant CAD in patients with no regional wall motion abnormalities and preserved LVEF
El Mahdiui et al.	Global left ventricular myocardial work efficiency in healthy individuals and patients with cardiovascular disease	2019	Journal of the American Society of Echocardiography	120	Global LV myocardial work efficiency is significantly reduced in patients after myocardial infarction or with HFrEF
Przewlocka-Kosmala et al.	Usefulness of myocardial work measurement in the assessment of left ventricular systolic reserve response to spironolactone in heart failure with preserved ejection fraction	2019	European Heart Journal	114	GCW is a better determinant of exercise capacity in HFpEF than GLS
Galli et al.	Myocardial constructive work is impaired in hypertrophic cardiomyopathy and predicts left ventricular fibrosis	2019	Ecocardiography	102	In contrast to LVEF, GCW is significantly reduced in HCM and is associated with LV fibrosis
Chan et al.	A new approach to assess myocardial work by non-invasive left ventricular pressure–strain relations in hypertension and dilated cardiomyopathy	2019	European Heart Journal	74	Non-invasive MW assessment leads to better comprehension of the interaction between LV remodeling and increased wall stress under different loading conditions
Schrub et al.	Myocardial work is a predictor of exercise tolerance in patients with dilated cardiomyopathy and left ventricular dyssynchrony	2020	The International Journal of Cardiovascular Imaging	51	Septal work efficiency is the only predictor of exercise capacity in patients with dilated cardiomyopathy and left ventricular dyssynchrony
Clemmensen et al.	Left ventricular pressure-strain–derived myocardial work at rest and during exercise in patients with cardiac amyloidosis	2020	Journal of the American Society of Echocardiography	155	Patients with cardiac amyloidosis have a significant reduction in GWI and GWE compared to healthy controls
Clemmensen et al.	Prognostic implications of left ventricular myocardial work indices in cardiac amyloidosis	2020	European Heart Journal Cardiovascular Imaging	100	In patients with cardiac amyloidosis, GWI and apical-to-basal segmental work ratio can better predict MACE and all-cause mortality than other echocardiographic parameters
Monsour et al.	Value of myocardial work for assessment of myocardial adaptation to increased afterload in patients with high blood pressure at peak exercise	2020	The International Journal of Cardiovascular Imaging	81	In patients with high blood pressure at peak exercise, GWI, GCW, and GWW increased significantly while GWE remained constant
Papadopoulos et al.	MitraClip and left ventricular reverse remodeling: a strain imaging study	2020	ESC Heart Failure	86	Preserved GLS and GCW values appear to be associated with LV reverse remodeling post intervention
Bouali et al.	Prognostic usefulness of myocardial work in patients with heart failure and reduced ejection fraction treated with sacubitril/valsartan	2020	The American Journal of Cardiology	79	Sacubitril/valsartan therapy is associated with a significant improvement in CW and WE. Assessment of CW before treatment can predict MACEs
Gonçovales et al.	Myocardial improvement after sacubitril/valsartan therapy: a new echocardiographic parameter for a new treatment	2020	Journal of Cardiovascular Medicine	42	Treatment with sacubitril/valsartan is related with an increase in global CW and WE as well as with reverse remodeling
Hedwig et al.	Global Work Index correlates with established prognostic parameters of heart failure	2020	Echocardiography	51	GWI correlates with known prognostic markers of heart failure
Wang et al.	Incremental prognostic value of global myocardial work over ejection fraction and global longitudinal strain in patients with heart failure and reduced ejection fraction	2020	European Heart Journal Cardiovascular Imaging	508	GMW is a better prognosticator than LVEF and GLS in HFrEF
Tomoaia et al.	Global work index by non-invasive pressure-strain loops: a novel parameter to assess left ventricular performance in the early stages of heart failure with preserved or mid-range ejection fraction after acute myocardial infarction	2021	Medical Ultrasonography	49	Non-invasive myocardial work estimation delivers further information about LV function in contrast to LVEF and GLS in patients after acute myocardial infarction at early stages of HFpEF/HFmrEF

*MWI* myocardial work index, *CAD* coronary artery disease, *HFrEF* heart failure with reduced ejection fraction, *WE* myocardial work efficiency, *MACEs* major adverse cardiac events, *GCW* global constructive myocardial work, *HFpEF* heart failure with preserved ejection fraction, *GLS* global longitudinal strain, *CW* constructive work, *GWI* global myocardial work index, *LVEF* left ventricular ejection fraction, *HCM* hypertrophic cardiomyopathy, *MW* myocardial work, *GWE* global myocardial work efficiency, *GWW* global myocardial wasted work

### Chronic heart failure

According to the current ESC Heart Failure Guidelines from 2016, heart failure represents a clinical diagnosis, which is characterized by typical symptoms and signs as well as increases of natriuretic peptide (BNP or NT-proBNP) [40].

Furthermore, echocardiographic determination of left ventricular function is necessary for the diagnosis of heart failure. Currently, left ventricular ejection fraction (LVEF) and also the assessment of left ventricular filling pressure by using the ratio of early transmitral flow and myocardial relaxation (E/e′ ratio) are the recommended parameters of choice [40].

The prognostic accuracy of LVEF, while significant in the circumstance of an LVEF < 40%, appears to be low in the case of HFpEF [74]. Additionally, LVEF is known to be significantly load dependent.

Today, myocardial strain measurements are well implemented in the daily clinical routine offering more precise, reproducible, and comprehensive information regarding LV mechanics and function. Clinical implications of myocardial strain assessment are diverse.

Notably, global longitudinal strain (GLS) was shown to be associated with outcome in symptomatic heart failure patients with reduced and preserved LVEF, and furthermore a stronger predictor of outcome than LVEF, especially in patients with preserved LVEF [75–78].

However, like LVEF, strain parameters prove to be dependent on afterload, resulting in a possible misinterpretation of the true contractile function [33, 79].

The estimation of myocardial work fixes this weakness by implementing the estimated LV pressure as described earlier.

In the setting of HFrEF, Wang et al. could show that global myocardial work (GMW) was a better prognosticator than both GLS and LVEF, where reduced values of GMW are significantly associated with death or poor outcome [80].

In a group of patients with acute myocardial infarction and heart failure with preserved or mid-range ejection fraction, it was shown that both GLS and Global Myocardial Work Index (GWI) are reduced in the majority of individuals. Some patients presented normal GWI despite abnormal GLS, emphasizing the importance of implementing blood pressure in the assessment of myocardial function [81].

Moreover, global constructive work (GCW) has been proven to be a better estimate of LV contractile response to physical effort, and hence a better measure of exercise capacity, in HFpEF than GLS. Its exertional increase in patients treated for 6 months with spironolactone is considered to be associated with improvement in functional capacity [82].

**Fig. 6:**
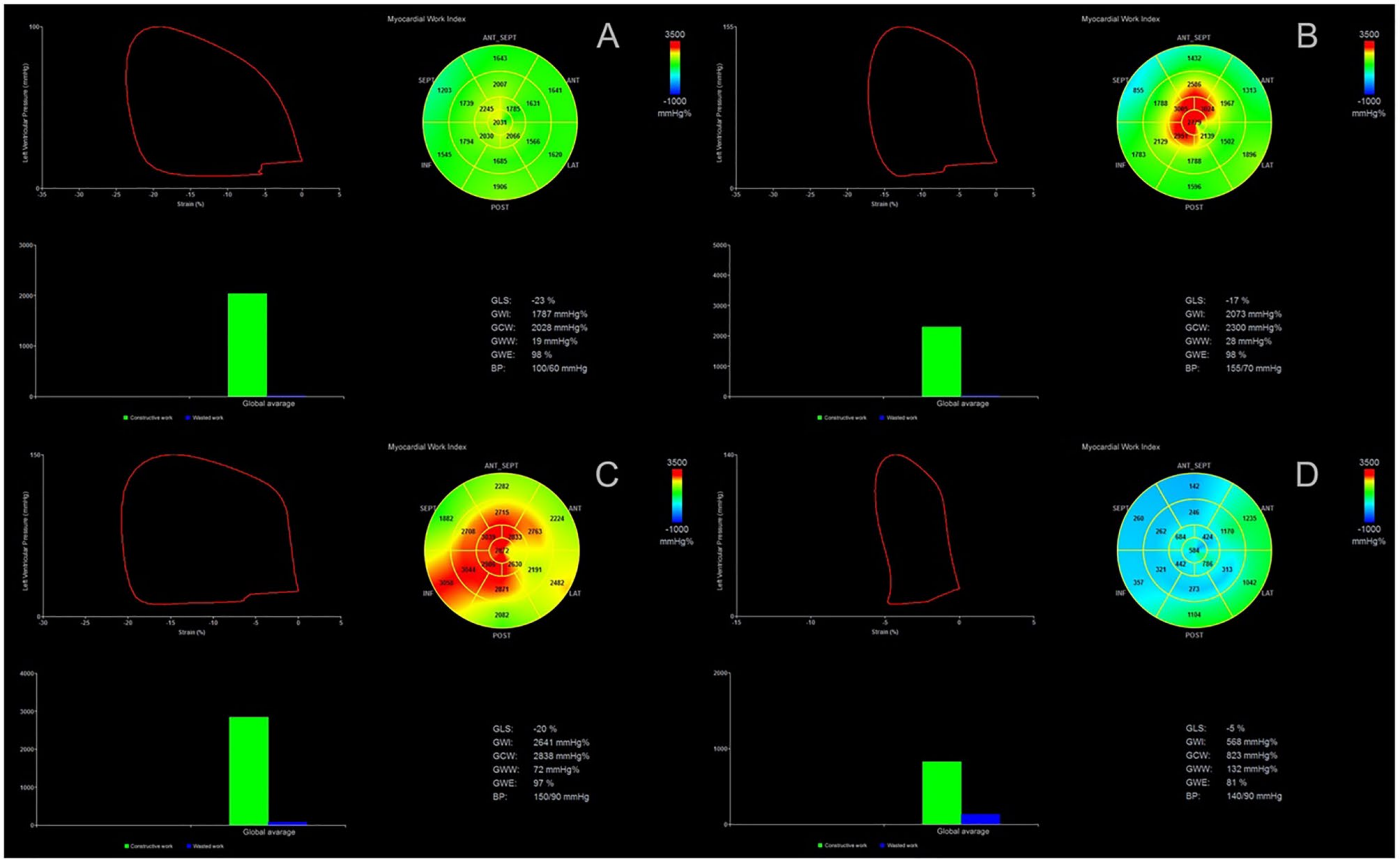
Measurement of myocardial work indices by 2D echocardiography; representative pressure strain–loops, bull’s eye plots of myocardial work index, and bar graphs showing GCW and GWW in control subjects (**A**) and subjects with HFmrEF (**B**), HFpEF (**C**), and HFrEF (**D**). GCW global constructive work, GWW global wasted work, HFmrEF heart failure with mid-range ejection fraction, HFpEF heart failure with preserved ejection fraction, HFrEF heart failure with reduced ejection fraction

Heart failure patients with reduced ejection fraction treated with sacubitril/valsartan showed signs of LV reverse remodeling by common echocardiographic parameters as well as a significant improvement of constructive work and myocardial work efficiency during a follow up of 12 months. Wasted work, on the contrary, did not appear to be greatly affected [83, 84]. Also, GCW could predict long-term outcome in patients with HFrEF receiving sacubitril/valsartan. Not only was a GCW ≤ 910 mmHg at baseline associated with a more advanced disease state, higher values for LV end-diastolic and end-systolic volume and more reduced LVEF but also a significant predictor of major adverse cardiac events (MACEs) before start of therapy [83]. Figure 6 demonstrates how changes in pressure-strain curves according to the LVEF range can be easily derived through the non-invasive assessment of MW by STE.

Hedwig et al. described the relation between global work index (GWI) and known prognostic parameters of echocardiography (GLS and LVEF), cardiopulmonary exercise test (Peak O_2_ consumption and VE/VCO_2_ slope), and NT-pro-BNP in patients with heart failure [85]. In particular, a GWI < 500 mmHg% could predict significant left ventricular remodeling, impaired LVEF, low exercise capacity, and high NT-pro-BNP levels, indicating dismal prognosis [85] (Table 3).

### Further applications of non-invasive myocardial work

Recently, Chan et al. described various patterns of non-invasively assessed myocardial work by pressure-strain analysis in healthy patients and in those suffering from arterial hypertension or dilated cardiomyopathies [86]. Especially patients suffering from arterial hypertension presented exciting results: In comparison to the control group, high systolic blood pressure (> 160 mmHg) led to a significant increase of GWI with no changes in MW efficiency, whereas GLS showed normal results [86]. These results confirm that usual STE-parameters as GLS are not able to reflect the increased cardiac energy demand to counteract increased afterload. Besides, in the cohorts of ischemic and not-ischemic dilated cardiomyopathy, there was a significant reduction of GWI and GWE due to an increase in wasted myocardial work and a reduction of constructive myocardial work [86].

Schrub et al. could also show in patients with dilated cardiomyopathy that the presence of left ventricular dyssynchrony assessed by echocardiography as septal flash or apical rocking leads to a significant reduction to global myocardial work efficiency due to an increase of wasted work [87]. These findings were especially present in the myocardial septum. Furthermore, the septal work efficiency was the only predictor of exercise capacity (VO_2_ peak) in these patients [87].

Galli et al. were the first to investigate pressure-strain analysis in patients with non-obstructive hypertrophic cardiomyopathy [35]. Here, global constructive work appeared to be significantly impaired in comparison to healthy individuals, despite no significant changes in LVEF [35]. At a multivariable regression analysis, GCW emerged as the main predictor of LV fibrosis assessed by late gadolinium enhancement [35]. Furthermore, GCW correlated with VO_2_ peak assessed by cardiopulmonal exercise testing, favoring GCW as an indirect, easily accessible measure of exercise capacity in patients with non-obstructive HCM [35].

Mansour et al. studied the variations of MW indices during stress echocardiography in patients at peak exercise with high systolic blood pressure values of > 180 mmHg [88]. They could confirm the results by Chan et al. [86], showing an increase of GCW, GWW, and GWI independent of peak GLS values at peak exercise, whereas GWE remained relatively preserved [88]. Furthermore, Mansour et al. investigated the contribution of the apex to MW. Here, the work done by the apex increased with peak exercise, emphasizing how the work done by the apex counteracts the energy loss measured by the GWW, especially in patients with SBP > 180 mmHg [88].

Non-invasive myocardial work has also been investigated in patients with cardiac amyloidosis at rest and during exercise by Clemmensen et al. [89]. These patients had significantly reduced measures of GWI and GWE at rest in comparison to healthy controls, especially pronounced in the basal segments of the myocardium. Furthermore, the myocardial work index reserve is significantly reduced in patients with cardiac amyloidosis in comparison to healthy individuals and correlates moderately with exercise capacity [89]. Another study by Clemmensen et al. could show that in patients with cardiac amyloidosis, GWI and the apical-to-segmental work ratio were able to predict MACE and all-cause mortality in these patients [90]. The new myocardial work indices outperformed GLS in predicting outcome, whereas GLS is already known to be more reliable than traditional echocardiographic parameters in the case of cardiac amyloidosis [90] (Table 3).

## Discussion

### Reference values of myocardial work and correlation with standard/advanced 2DE parameters

The NORRE (Normal Reference Ranges for Echocardiography) study, the first related multicenter study, investigated a large population of healthy individuals (*n* = 226) over a wide range of ages and delivered current normal reference values of non-invasive myocardial work indices [91]. Only in univariable analysis, GWI and GCW were age dependent. Merely in woman, both GWI and GCW increased with age due to an age-dependent significant increase of both systolic and diastolic blood pressure only in the female subgroup [91]. Moreover, multivariable analysis showed a gender and age independent correlation of both GWI and GCW only with systolic blood pressure [91]. Also, solely in univariable analysis, GWW and GWE showed independently of age minor as well as increased values in female individuals, respectively [91]. In conclusion, MW indices were not strongly dependent on age or gender. However, GWI and GWC were clearly associated with systolic blood pressure.

In a following sub study of the NORRE study, Manganaro et al. investigated the correlations between the new indices of non-invasive MW with main measures of systolic and diastolic function [92]. In multivariable analysis, the correlation between MW indices and LV size was not sufficiently significant. In fact, GWW and GWE poorly correlated with end systolic volume, while GWI and GCW were poorly correlated to LV mass adjusted to body surface area (BSA). These findings were not confirmed in multivariable analysis so that their relevance should be regarded as limited [92]. These results probably rely on the actual study population that only included healthy subjects, where LV size and consequentially parameters of cardiac function like MW indices are in normal ranges.

Similar considerations can be inferred when analyzing diastolic function: GWI and GCW correlated with LA size and E/E′ ratio only in univariable analysis, leading to the consideration that MW and diastolic parameters are weakly associated [92]. Here, the Tei index made an exception, which proved to be significantly correlated with both GWW and GWE [92]. Moreover, both GWI and GCW were as expected associated with GLS, LVEF, and SBP as well as with global radial strain. In addition, GCW was also significantly correlated with global circumferential strain [92].

### Intra- and interobserver variability

Several studies have tested the reproducibility of non-invasive myocardial work indices, showing good results regarding both intra- and inter-observer variability [22, 34, 36, 38, 56, 85, 91]

### Limitations of non-invasive pressure estimation

Naturally, the LV pressure estimation cannot fully reproduce direct invasive measurements. Hence, pressure strain analysis comes with certain limitations which are essential for the understanding of this new method. Myocardial work by pressure strain analysis has to be understood as an index of cardiac work, as work is defined as the product of force and time. Pressure-strain analysis delivers a surrogate of the myocardial work performed by each segment, as LV pressure does not entirely explain each segment’s force development [22]. When comparing left ventricles with different dimensions as in the study by Chan et al., myocardial work could be underestimated in dilated hearts due to their higher wall stress for each given LV-pressure [93, 94]. It is shown that the estimation of LV pressure itself is imprecise, leading to a bias of overestimation of the regional pressure-strain area [13]. Interestingly, Hubert et al. could show that the estimated global myocardial work parameters correlate very well with the invasively measured cardiac work [13].

In conditions of specific valvular pathologies, for example, aortic stenosis or left ventricular output obstruction, the estimation of LV peak pressure by measuring peripheral systolic arterial pressure cannot be used. The pressure gradient across the ventricle and the peripheral artery would lead to a false estimation of the actual LV pressure [22]. Regarding further disadvantages in comparison to invasive measurements, pressure-strain loop analysis is not able to deliver information about LV diastolic pressure or maximal rate of rise or fall of LV pressure [22].

Furthermore, since single blood pressure measurements can be very variable in the same patient during the day, this variability could eventually affect the assessment of myocardial work. The impact of taking a 24-h blood pressure measurement into account for the calculation of myocardial work needs further investigation. Also, strain analysis by echocardiography inherently depends on image quality which might have an impact on the feasibility of this method for some patients. In patients with cardiac arrhythmias, e.g., atrial fibrillation, GLS by STE might not be accurately assessable, questioning the estimation of myocardial work indices in these patients. Additionally, the estimation of myocardial work is software dependent and might differ among different vendors as it does for GLS [95]. Currently, the only software package available is provided by GE (Echopac V.202, GE). Boe et al. suggested that future improvements to the method, potentially by using 3D echocardiography, should include information about LV geometry, wall thickness, and local radii allowing the measurement of wall-stress instead of pressure [94]. Regarding the echocardiographic reference ranges, further studies in larger cohorts and different cardiac pathologies are needed. If the current results are valid for non-Caucasian European individuals is still uncertain [91].

### Prognostic value of non-invasive myocardial work assessment: contemporary and upcoming challenges

As abovementioned, non-invasive assessment of myocardial work has been investigated in different cardiac conditions, ranging from coronary artery disease, including patients with NSTEMI, to LV-dyssynchrony, arterial hypertension, cardiac amyloidosis, and dilated and hypertrophic cardiomyopathy [33–36, 38, 56, 57, 82, 86–89]. However, to date, there are only a few small-scale studies which investigated the prognostic relevance of non-invasively assessed myocardial work, namely, studies on CRT and heart failure [37, 58, 83, 85]. GCW and GWW, respectively as measures of cardiac reserve and energy loss, provide additional information to commonly used echocardiographic parameters in identifying CRT responders. The prospective, observational Contractile Reserve in Dyssynchrony (CRID) study investigated if wasted work can predict response to CRT in 210 participants who fulfill the indications of a CRT-device implantation. The study was completed in December 2018, and the results are yet to be published [96]. In another ongoing study by Jens-Uwe Voigt et al. entitled “Myocardial Work and Metabolism in CRT (WORK-CRT),” non-invasive measures of LV mechanical dyssynchrony (e.g., apical rocking) will be examined to determine their predictive value in CRT response [97].

A further randomized interventional trial, “Early rhBNP on Myocardial Work in Patients With STEMI,” by Song Ding et al. started in November 2019 and is set to end in September 2022, with an estimated number of 200 enrolled patients [98]. This study aims to assess the impact of early intracoronary injection and ongoing intravenous infusion of recombinant human BNP (rhBNP) over 72 h on myocardial work in patients with anterior STEMI after percutaneous coronary intervent [98]. The impact of non-invasively estimated myocardial work for the prediction of mortality in patients with acute myocardial infarction has yet to be determined. So far, pressure-strain analysis has not been investigated in patients with ST-segment elevation myocardial infarction.

In patients with cardiogenic shock, measures of myocardial work were shown to be the strongest predictors of intra-hospital mortality, especially the cardiac power output (CPO) [99]. CPO is a parameter of external cardiac work, calculated as the product of cardiac output and mean aortic pressure divided by 451. In a recently published study of our group, we could show that CPO precisely reflects left ventricular stroke work per minute over a broad scope of inotropic states [100]. Here, CPO was measured by right heart catheterization (RHC) as well as invasive arterial pressure which were correlated with LV SW min^−1^ measured via the conductance method, the invasive gold-standard. LVEF did not reflect myocardial work at all [100]. However, the use of RHC is controversial in critically ill patients [101]. The non-invasive estimation of myocardial work indices could be very useful to monitor cardiac function and inotropic interventions in the ICU setting.

Another interesting outlook is related to the field of trans-catheter valve repair. Papadopoulos et al. conducted a retrospective case–control study to evaluate left ventricular global longitudinal strain and myocardial work indices in patients with heart failure and mitral regurgitation 1 year after transcatheter mitral-valve repair compared to patients treated with optimal medical treatment [102]. Preserved baseline GLS and GCW appeared to be able to predict reduction in LVEDV and LVESV, respectively, after successful MV repair proving to be potential new markers of positive LV remodeling, which could allow better patients’ selection for this procedure [102]. In addition, patients who were treated with a MitraClip® showed stable GWE as well as improved GCW and GWI at follow-up, reflecting better cardiac energetics in contrast to patients treated with optical medical treatment [102].

## Conclusions

The ability to assess myocardial work non-invasively broadens the currently available techniques for evaluating cardiac function. The adjustment to loading conditions leads to the implementation of critical hemodynamic concepts in the daily assessment of cardiac function. Further studies focusing on the prognostic impact of non-invasive myocardial work are needed.

## References

[1] Zaret BL, Wackers FJT, Terrin ML (1995). Value of radionuclide rest and exercise left ventricular ejection fraction in assessing survival of patients after thrombolytic therapy for acute myocardial infarction: results of thrombolysis in myocardial infarction (TIMI) phase II study. J Am Coll Cardiol.

[2] Burns RJ, Gibbons RJ, Yi Q (2002). The relationships of left ventricular ejection fraction, end-systolic volume index and infarct size to six-month mortality after hospital discharge following myocardial infarction treated by thrombolysis. J Am Coll Cardiol.

[3] Nicolosi GL, Latini R, Marino P (1996). The prognostic value of predischarge quantitative two-dimensional echocardiographic measurements and the effects of early lisinopril treatment on left ventricular structure and function after acute myocardial infarction in the GISSI-3 trial. Eur Heart J.

[4] Christian TF, Behrenbeck T, Gersh BJ, Gibbons RJ (1991). Relation of left ventricular volume and function over one year after acute myocardial infarction to infarct size determined by technetium-99m sestamibi. Am J Cardiol.

[5] Galli E, Lancellotti P, Sengupta PP, Donal E (2014). LV mechanics in mitral and aortic valve diseases :value of functional assessment beyond ejection fraction. JACC Cardiovasc Imaging.

[6] Plana JC, Galderisi M, Barac A (2014). Expert consensus for multimodality imaging evaluation of adult patients during and after cancer therapy: a report from the American Society of Echocardiography and the European Association of Cardiovascular Imaging. Eur Heart J Cardiovasc Imaging.

[7] di Bella G, Minutoli F, Pingitore A (2011). Endocardial and epicardial deformations in cardiac amyloidosis and hypertrophic cardiomyopathy-2-D feature strain echocardiography. Circ J.

[8] Zito C, Manganaro R, Khandheria B (2015). Usefulness of left atrial reservoir size and left ventricular untwisting rate for predicting outcome in primary mitral regurgitation. Am J Cardiol.

[9] Donal E, Thebault C, Oconnor K (2011). Impact of aortic stenosis on longitudinal myocardial deformation during exercise. Eur J Echocardiogr.

[10] Kasner M, Aleksandrov A, Escher F (2017). Multimodality imaging approach in the diagnosis of chronic myocarditis with preserved left ventricular ejection fraction (MCpEF): The role of 2D speckle-tracking echocardiography. Int J Cardiol.

[11] Tschöpe C, Senni M (2020). Usefulness and clinical relevance of left ventricular global longitudinal systolic strain in patients with heart failure with preserved ejection fraction. Heart Fail Rev.

[12] Mor-Avi V, Patel MB, Maffessanti F (2018). Fusion of three-dimensional echocardiographic regional myocardial strain with cardiac computed tomography for noninvasive evaluation of the hemodynamic impact of coronary stenosis in patients with chest pain. J Am Soc Echocardiogr.

[13] Hubert A, Le Rolle V, Leclercq C (2018). Estimation of myocardial work from pressure–strain loops analysis: an experimental evaluation. Eur Hear J - Cardiovasc Imaging.

[14] Burkhoff D, Mirsky I, Suga H (2005). Assessment of systolic and diastolic ventricular properties via pressure-volume analysis: a guide for clinical, translational, and basic researchers. Am J Physiol Heart Circ Physiol.

[15] Kass DA, Yamazaki T, Burkhoff D (1986). Determination of left ventricular end-systolic pressure-volume relationships by the conductance (volume) catheter technique. Circulation.

[16] Kass DA, Midei M, Graves W (1988). Use of a conductance (volume) catheter and transient inferior vena caval occlusion for rapid determination of pressure-volume relationships in man. Cathet Cardiovasc Diagn.

[17] Westermann D, Kasner M, Steendijk P (2008). Role of left ventricular stiffness in heart failure with normal ejection fraction. Circulation.

[18] Suga H (1990). Ventricular energetics. Physiol Rev.

[19] Suga H (1979). Total mechanical energy of a ventricle model and cardiac oxygen consumption. Am J Physiol.

[20] Baan J, van der Velde ET, de Bruin HG (1984). Continuous measurement of left ventricular volume in animals and humans by conductance catheter. Circulation.

[21] Kasner M, Sinning D, Burkhoff D, Tschöpe C (2015). Diastolic pressure-volume quotient (DPVQ) as a novel echocardiographic index for estimation of LV stiffness in HFpEF. Clin Res Cardiol.

[22] Russell K, Eriksen M, Aaberge L (2012). A novel clinical method for quantification of regional left ventricular pressure–strain loop area: a non-invasive index of myocardial work. Eur Heart J.

[23] Morris JJ, Pellom GL, Murphy CE (1987). Quantification of the contractile response to injury: assessment of the work-length relationship in the intact heart. Circulation.

[24] Safwat A, Leone BJ, Norris RM, Foëx P (1991). Pressure-length loop area: its components analyzed during graded myocardial ischemia. J Am Coll Cardiol.

[25] Rushmer RF, Franklin DL, Ellis RM (1956). Left ventricular dimensions recorded by sonocardiometry. Circ Res.

[26] Miyamoto MI, Kim CS, Guerrero JL (1999). Ventricular pressure and dimension measurements in mice. Lab Anim Sci.

[27] Pasipoularides A, Shu M, Shah A (2002). Right ventricular diastolic function in canine models of pressure overload, volume overload, and ischemia. Am J Physiol Circ Physiol.

[28] Leraand S, Kiil F (1969). Local dimensional changes of the myocardium measured by ultrasonic technique. Scand J Clin Lab Invest.

[29] Delhaas T, Arts T, Prinzen FW, Reneman RS (1998). Estimates of regional work in the canine left ventricle. Prog Biophys Mol Biol.

[30] Skulstad H, Edvardsen T, Urheim S (2002). Postsystolic shortening in ischemic myocardium: active contraction or passive recoil?. Circulation.

[31] Urheim S, Rabben SI, Skulstad H (2005). Regional myocardial work by strain Doppler echocardiography and LV pressure: a new method for quantifying myocardial function. Am J Physiol - Hear Circ Physiol.

[32] Russell K, Eriksen M, Aaberge L (2013). Assessment of wasted myocardial work: a novel method to quantify energy loss due to uncoordinated left ventricular contractions. Am J Physiol Circ Physiol.

[33] Boe E, Russell K, Eek C (2015). Non-invasive myocardial work index identifies acute coronary occlusion in patients with non-ST-segment elevation-acute coronary syndrome. Eur Hear J - Cardiovasc Imaging.

[34] Edwards NFA, Scalia GM, Shiino K (2019). Global myocardial work is superior to global longitudinal strain to predict significant coronary artery disease in patients with normal left ventricular function and wall motion. J Am Soc Echocardiogr.

[35] Galli E, Vitel E, Schnell F (2019). Myocardial constructive work is impaired in hypertrophic cardiomyopathy and predicts left ventricular fibrosis. Echocardiography.

[36] Galli E, Leclercq C, Fournet M (2018). Value of myocardial work estimation in the prediction of response to cardiac resynchronization therapy. J Am Soc Echocardiogr.

[37] Galli E, Hubert A, Le Rolle V (2019). Myocardial constructive work and cardiac mortality in resynchronization therapy candidates. Am Heart J.

[38] Galli E, Leclercq C, Hubert A (2018). Role ofmyocardial constructive work in the identification of responders to CRT. Eur Heart J Cardiovasc Imaging.

[39] Wells G, Parkash R, Healey JS (2011). Cardiac resynchronization therapy: a meta-analysis of randomized controlled trials. CMAJ.

[40] Ponikowski P, Voors AA, Anker SD (2016). 2016 ESC guidelines for the diagnosis and treatment of acute and chronic heart failure. Eur Heart J.

[41] Chung ES, Leon AR, Tavazzi L (2008). Results of the Predictors of Response to CRT (PROSPECT) trial. Circulation.

[42] Ypenburg C, van Bommel RJ, Borleffs CJW (2009). Long-term prognosis after cardiac resynchronization therapy is related to the extent of left ventricular reverse remodeling at midterm follow-up. J Am Coll Cardiol.

[43] van Bommel RJ, Tanaka H, Delgado V (2010). Association of intraventricular mechanical dyssynchrony with response to cardiac resynchronization therapy in heart failure patients with a narrow QRS complex. Eur Heart J.

[44] Yu C-M, Fung W-H, Lin H (2003). Predictors of left ventricular reverse remodeling after cardiac resynchronization therapy for heart failure secondary to idiopathic dilated or ischemic cardiomyopathy. Am J Cardiol.

[45] Søgaard P, Egeblad H, Kim WY (2002). Tissue Doppler imaging predicts improved systolic performance and reversed left ventricular remodeling during long-term cardiac resynchronization therapy. J Am Coll Cardiol.

[46] Arshad A, Moss AJ, Foster E (2011). Cardiac resynchronization therapy is more effective in women than in men: the MADIT-CRT (Multicenter Automatic Defibrillator Implantation Trial with Cardiac Resynchronization Therapy) trial. J Am Coll Cardiol.

[47] McLeod CJ, Shen W-K, Rea RF (2011). Differential outcome of cardiac resynchronization therapy in ischemic cardiomyopathy and idiopathic dilated cardiomyopathy. Hear Rhythm.

[48] Park MY, Altman RK, Orencole M (2012). Characteristics of responders to cardiac resynchronization therapy: the impact of echocardiographic left ventricular volume. Clin Cardiol.

[49] Reant P, Zaroui A, Donal E et al (2010) Identification and characterization of super-responders after cardiac resynchronization therapy. Am J Cardiol 105:1327–1335. 10.1016/j.amjcard.2009.12.058 10.1016/j.amjcard.2009.12.058 20403487

[50] Ciampi Q, Pratali L, Citro R (2009). Identification of responders to cardiac resynchronization therapy by contractile reserve during stress echocardiography. Eur J Heart Fail.

[51] Parsai C, Bijnens B, Sutherland GR (2009). Toward understanding response to cardiac resynchronization therapy: left ventricular dyssynchrony is only one of multiple mechanisms. Eur Heart J.

[52] Brunet-Bernard A, Maréchaux S, Fauchier L (2014). Combined score using clinical, electrocardiographic, and echocardiographic parameters to predict left ventricular remodeling in patients having had cardiac resynchronization therapy six months earlier. Am J Cardiol.

[53] Lafitte S, Reant P, Zaroui A (2009). Validation of an echocardiographic multiparametric strategy to increase responders patients after cardiac resynchronization: a multicentre study. Eur Heart J.

[54] Delgado V, van Bommel RJ, Bertini M (2011). Relative merits of left ventricular dyssynchrony, left ventricular lead position, and myocardial scar to predict long-term survival of ischemic heart failure patients undergoing cardiac resynchronization therapy. Circulation.

[55] Lim P, Buakhamsri A, Popovic ZB (2008). Longitudinal strain delay index by speckle tracking imaging: a new marker of response to cardiac resynchronization therapy. Circulation.

[56] Vecera J, Penicka M, Eriksen M (2016). Wasted septal work in left ventricular dyssynchrony: a novel principle to predict response to cardiac resynchronization therapy. Eur Hear J - Cardiovasc Imaging.

[57] Zhu M, Chen H, Fulati Z (2019). The value of left ventricular strain–volume loops in predicting response to cardiac resynchronization therapy. Cardiovasc Ultrasound.

[58] van der Bijl P, Vo NM, Kostyukevich MV (2019). Prognostic implications of global, left ventricular myocardial work efficiency before cardiac resynchronization therapy. Eur Hear J - Cardiovasc Imaging.

[59] Duchenne J, Aalen JM, Cvijic M (2020). Acute redistribution of regional left ventricular work by cardiac resynchronization therapy determines long-term remodelling. Eur Hear J - Cardiovasc Imaging.

[60] Kostyukevich MV, van der Bijl P, Vo NM (2020). Regional left ventricular myocardial work indices and response to cardiac resynchronization therapy. JACC Cardiovasc Imaging.

[61] Sorajja P, Gersh BJ, Cox DA (2010). Impact of delay to angioplasty in patients with acute coronary syndromes undergoing invasive management. analysis from the ACUITY (Acute Catheterization and Urgent Intervention Triage strategY) trial. J Am Coll Cardiol.

[62] Jneid H, Anderson JL, Wright RS (2012). 2012 ACCF/AHA focused update of the guideline for the management of patients with unstable angina/non–st-elevation myocardial infarction (updating the 2007 guideline and replacing the 2011 focused update). Circulation.

[63] Choi J-O, Cho SW, Bin SY (2009). Longitudinal 2D strain at rest predicts the presence of left main and three vessel coronary artery disease in patients without regional wall motion abnormality. Eur J Echocardiogr.

[64] Montgomery DE, Puthumana JJ, Fox JM, Ogunyankin KO (2012). Global longitudinal strain aids the detection of non-obstructive coronary artery disease in the resting echocardiogram. Eur Heart J Cardiovasc Imaging.

[65] Yingchoncharoen T, Agarwal S, Popović ZB, Marwick TH (2013). Normal ranges of left ventricular strain: a meta-analysis. J Am Soc Echocardiogr.

[66] Tsai W-C, Liu Y-W, Huang Y-Y (2010). Diagnostic value of segmental longitudinal strain by automated function imaging in coronary artery disease without left ventricular dysfunction. J Am Soc Echocardiogr.

[67] Dahlslett T, Karlsen S, Grenne B (2014). Early assessment of strain echocardiography can accurately exclude significant coronary artery stenosis in suspected non-ST-segment elevation acute coronary syndrome. J Am Soc Echocardiogr.

[68] Sjøli B, Ørn S, Grenne B (2009). Diagnostic capability and reproducibility of strain by Doppler and by speckle tracking in patients with acute myocardial infarction. JACC Cardiovasc Imaging.

[69] Shimoni S, Gendelman G, Ayzenberg O (2011). Differential effects of coronary artery stenosis on myocardial function: the value of myocardial strain analysis for the detection of coronary artery disease. J Am Soc Echocardiogr.

[70] Winter R, Jussila R, Nowak J, Brodin L-A (2007). Speckle tracking echocardiography is a sensitive tool for the detection of myocardial ischemia: a pilot study from the catheterization laboratory during percutaneous coronary intervention. J Am Soc Echocardiogr.

[71] Hanekom L, Cho G-Y, Leano R (2007). Comparison of two-dimensional speckle and tissue Doppler strain measurement during dobutamine stress echocardiography: an angiographic correlation. Eur Heart J.

[72] Reimer KA, Jennings RB (1979). The “wavefront phenomenon” of myocardial ischemic cell death. II. Transmural progression of necrosis within the framework of ischemic bed size (myocardium at risk) and collateral flow. Lab Invest.

[73] El Mahdiui M, van der Bijl P, Abou R (2019). Global left ventricular myocardial work efficiency in healthy individuals and patients with cardiovascular disease. J Am Soc Echocardiogr.

[74] Solomon SD, Anavekar N, Skali H (2005). Influence of ejection fraction on cardiovascular outcomes in a broad spectrum of heart failure patients. Circulation.

[75] Huang W, Chai SC, Lee SGS (2017). Prognostic factors after index hospitalization for heart failure with preserved ejection fraction. Am J Cardiol.

[76] Sengeløv M, Jørgensen PG, Jensen JS (2015). Global longitudinal strain is a superior predictor of all-cause mortality in heart failure with reduced ejection fraction. JACC Cardiovasc Imaging.

[77] Kalam K, Otahal P, Marwick TH (2014). Prognostic implications of global LV dysfunction: a systematic review and meta-analysis of global longitudinal strain and ejection fraction. Heart.

[78] Stanton T, Leano R, Marwick TH (2009). Prediction of all-cause mortality from global longitudinal speckle strain. Circ Cardiovasc Imaging.

[79] Donal E, Bergerot C, Thibault H (2009). Influence of afterload on left ventricular radial and longitudinal systolic functions: a two-dimensional strain imaging study. Eur J Echocardiogr J Work Gr Echocardiogr Eur Soc Cardiol.

[80] Wang CL, Chan YH, Wu VCC et al (2020) Incremental prognostic value of global myocardial work over ejection fraction and global longitudinal strain in patients with heart failure and reduced ejection fraction. Eur Hear J - Cardiovasc Imaging 348–356. 10.1093/ehjci/jeaa162 10.1093/ehjci/jeaa162 32820318

[81] Tomoaia R, Beyer RS, Zdrenghea D et al (2020) Global work index by non-invasive pressure-strain loops: a novel parameter to assess left ventricular performance in the early stages of heart failure with preserved or mid-range ejection fraction after acute myocardial infarction. Med Ultrason 23:62–69. 10.11152/mu-2672 10.11152/mu-2672 33220030

[82] Przewlocka-Kosmala M, Marwick TH, Mysiak A (2019). Usefulness of myocardial work measurement in the assessment of left ventricular systolic reserve response to spironolactone in heart failure with preserved ejection fraction. Eur Heart J Cardiovasc Imaging.

[83] Bouali Y, Donal E, Gallard A (2020). Prognostic usefulness of myocardial work in patients with heart failure and reduced ejection fraction treated by sacubitril/valsartan. Am J Cardiol.

[84] Valentim Gonçalves A, Galrinho A, Pereira-da-Silva T (2020). Myocardial work improvement after sacubitril-valsartan therapy: a new echocardiographic parameter for a new treatment. J Cardiovasc Med (Hagerstown).

[85] Hedwig F, Soltani S, Stein J (2020). Global work index correlates with established prognostic parameters of heart failure. Echocardiography.

[86] Chan J, Edwards NFA, Khandheria BK (2019). A new approach to assess myocardial work by non-invasive left ventricular pressure-strain relations in hypertension and dilated cardiomyopathy. Eur Heart J Cardiovasc Imaging.

[87] Schrub F, Schnell F, Donal E, Galli E (2019). Myocardial work is a predictor of exercise tolerance in patients with dilated cardiomyopathy and left ventricular dyssynchrony. Int J Cardiovasc Imaging.

[88] Mansour MJ, AlJaroudi W, Mansour L (2020). Value of myocardial work for assessment of myocardial adaptation to increased afterload in patients with high blood pressure at peak exercise. Int J Cardiovasc Imaging.

[89] Clemmensen TS, Eiskjær H, Mikkelsen F (2020). Left ventricular pressure-strain–derived myocardial work at rest and during exercise in patients with cardiac amyloidosis. J Am Soc Echocardiogr.

[90] Clemmensen TS, Eiskjær H, Ladefoged B et al (2020) Prognostic implications of left ventricular myocardial work indices in cardiac amyloidosis. Eur Hear J - Cardiovasc Imaging 1–10. 10.1093/ehjci/jeaa097 10.1093/ehjci/jeaa097 32529207

[91] Manganaro R, Marchetta S, Dulgheru R (2019). Echocardiographic reference ranges for normal non-invasive myocardial work indices: results from the EACVI NORRE study. Eur Heart J Cardiovasc Imaging.

[92] Manganaro R, Marchetta S, Dulgheru R (2019). Correlation between non-invasive myocardial work indices and main parameters of systolic and diastolic function: results from the EACVI NORRE study. Eur Hear J - Cardiovasc Imaging.

[93] Chan J, Edwards NFA, Khandheria BK (2019). A new approach to assess myocardial work by non-invasive left ventricular pressure–strain relations in hypertension and dilated cardiomyopathy. Eur Hear J - Cardiovasc Imaging.

[94] Boe E, Skulstad H, Smiseth OA (2019). Myocardial work by echocardiography: a novel method ready for clinical testing. Eur Heart J Cardiovasc Imaging.

[95] Farsalinos KE, Daraban AM, Ünlü S (2015). Head-to-head comparison of global longitudinal strain measurements among nine different vendors: the EACVI/ASE inter-vendor comparison study. J Am Soc Echocardiogr.

[96] US National Library of Medicine (2015) Contractile reserve in dyssynchrony: a novel principle to identify candidates for cardiac resynchronization therapy (CRID-CRT). In: ClinicalTrials.gov. https://clinicaltrials.gov/ct2/show/NCT02525185

[97] US National Library of Medicine (2015) Myocardial work and metabolism in CRT (WORK-CRT). In: ClinicalTrials.gov. https://clinicaltrials.gov/ct2/show/NCT02537782

[98] US National Library of Medicine (2019) The Impact of Early rhBNP on myocardial work in patients with anterior ST-segment elevation myocardial infarction after percutaneous coronary intervention. In: Clinical Trials.gov. https://clinicaltrials.gov/ct2/show/NCT04157868

[99] Fincke R, Hochman JS, Lowe AM (2004). Cardiac power is the strongest hemodynamic correlate of mortality in cardiogenic shock: a report from the SHOCK trial registry. J Am Coll Cardiol.

[100] Abawi D, Faragli A, Schwarzl M (2019). Cardiac power output accurately reflects external cardiac work over a wide range of inotropic states in pigs. BMC Cardiovasc Disord.

[101] Shah MR, Hasselblad V, Stevenson LW (2005). Impact of the pulmonary artery catheter in critically ill patients. JAMA.

[102] Papadopoulos K, Ikonomidis I, Chrissoheris M (2020). MitraClip and left ventricular reverse remodelling: a strain imaging study. ESC Hear Fail.

